# Effects of Latanoprost and Bimatoprost on the Expression of Molecules Relevant to Ocular Inflow and Outflow Pathways

**DOI:** 10.1371/journal.pone.0151644

**Published:** 2016-03-24

**Authors:** Xiaohong Li, Fen He, B’Ann T. Gabelt, Yun Wang, Suping Cai, Juanhui Cao, Ning Fan, Paul L. Kaufman, Xuyang Liu

**Affiliations:** 1 Department of Biopharmaceutics, Key Laboratory of Drug Targeting and Drug Delivery Systems, Ministry of Education, West China School of Pharmacy, Sichuan University, Chengdu, China; 2 State Key Laboratory of Biotherapy, West China Hospital, Sichuan University, Chengdu, China; 3 Department of Ophthalmology & Visual Sciences, University of Wisconsin-Madison, Madison, Wisconsin, United States of America; 4 Shenzhen Key Laboratory of Ophthalmology, Shenzhen Eye Hospital, Shenzhen University, Shenzhen, China; University of Iowa, UNITED STATES

## Abstract

**Background and Purpose:**

The intraocular pressure (IOP)-lowering and side effects in response to different prostaglandin F_2_α analogues can be variable, but, the underlying basis for this difference remains unknown. This study investigated the differential changes of cellular proteins relevant to IOP-lowering effects of latanoprost and bimatoprost.

**Methods:**

The human T lymphoblast (MOLT-3) cell line and immortalized human trabecular meshwork (iHTM) cells were studied by quantitative PCR and by immunofluorescence after treatment with either latanoprost or bimatoprost. New Zealand white rabbit eyes were treated topically with each agent and, following euthanasia, anterior segment tissues were studied with immunostaining.

**Results:**

In cultured MOLT-3 cells, mRNA expression of both c-fos and matrix metalloproteinase 9 increased significantly in response to each agent. In addition, there was little change in tissue inhibitor of metalloproteinase (TIMP)-3 mRNA, but a significant decrease in TIMP-4. Fibronectin mRNA in MOLT-3 cells was down-regulated with bimatoprost, but was up-regulated with latanoprost. Immunofluorescence analysis of iHTM cells showed that intracellular fibronectin was significantly decreased by bimatoprost, but was increased by latanoprost. Both latanoprost and bimatoprost increased mRNA expression of NF-кB p65 and decreased that of IкBα. Aquaporin-1 mRNA expression was significantly down-regulated by bimatoprost. Immunostaining also revealed a significant decrease of aquaporin-1 in the ciliary epithelium of New Zealand white rabbits after bimatoprost treatment.

**Conclusions:**

Similarities in protein expression produced by latanoprost and bimatoprost in vitro may be relevant to the mechanism for their IOP-lowering effects in vivo. Differences in fibronectin expression and in aquaporin-1 expression in response to each agent may contribute to variability in the IOP-lowering efficacy in some studies.

## Introduction

Prostaglandin F_2_α analogues (PGAs), including latanoprost, travoprost and bimatoprost, are considered as the first choice for the pharmaceutical treatment of glaucoma and ocular hypertension based on their effectiveness in lowering intraocular pressure (IOP) and few systemic side effects [1]. IOP lowering and side effects in response to different PGAs can be variable [2–5]. Some studies demonstrated that bimatoprost and latanoprost elicited similar IOP lowering responses, whereas in others bimatoprost appeared to be slightly more effective [6–9]. Some patients who were poorly or non-responsive to latanoprost responded well to bimatoprost [3]. No studies have reported that the reverse was true. The underlying basis for this difference remains unknown.

It is generally accepted that all PGAs exert their effects, both therapeutic and adverse, via their receptors. Latanoprost and travoprost interact with prostaglandin F receptors (FP). The receptor for bimatoprost is still controversial [10, 11]. There is experimental evidence suggesting that bimatoprost, different from the FP agonists latanoprost and travoprost, may act as a prostamide at its own receptor. Chen et al. reported that the human T lymphoblast (peripheral blood acute lymphoblastic leukemia, MOLT-3) cells expressed no FP or thromboxane A_2_ receptors (TP) based on qPCR analysis, and that bimatoprost exerted its effects independent of FP and TP receptors [11]. A so-called undefined receptor for bimatoprost might elicit different effects, therapeutic and adverse, from latanoprost. However, in a previous study we demonstrated that MOLT-3 cells expressed the FP receptor based on Western blot and mass spectrometry analyses [12]. Whether the effects of bimatoprost are independent of the FP receptor needs further investigation. We have hypothesized that the differential receptor selectivity of various PGAs to various prostanoid receptors, including FP and EP, might mediate their diversified effects [13, 14]. Some differential effects of the different PGAs might involve differential changes of cellular proteins that are elicited from the diversified receptor selectivity of various PGAs.

To understand the diversified effects of various PGAs, differential changes of cellular proteins, previously reported to be involved in IOP regulation or in possible mechanisms for the IOP lowering effects of PGAs, were investigated following 5-day PGA exposure of cells or tissues. Two PGAs, latanoprost, a representative PGA analog acting via the FP receptor, and bimatoprost, a prostamide mimetic whose receptor is still controversial [10, 11], were investigated. The targeted proteins included transcription factors c-fos, matrix metalloproteinases (MMPs) and their inhibitors, fibronectin, aquaporin-1 (AQP1), and nuclear factor-kappa B (NF-кB).

The IOP in any given eye is determined by the rate of aqueous production and the drainage of aqueous humor. In order to decrease IOP, most of the anti-glaucoma medications act either by decreasing the rate of aqueous humor production or by enhancing aqueous outflow, including the conventional trabecular meshwork (TM) outflow pathway and the unconventional uveoscleral pathway. Considering the reported similar effects of various PGAs on the unconventional uveoscleral pathway [15], we investigated the conventional TM pathway using immortalized human TM (iHTM) cells, and the aqueous humor inflow pathway in rabbit eyes, hoping to find some differential effects of the different PGAs. Once different effects of bimatoprost and latanoprost on mRNA expression were observed in MOLT-3 cells, the proteins in iHTM cells or in the anterior segments of the rabbit eyes were subsequently investigated.

## Materials and Methods

Sichuan University Institute Review Board approved this research.

### Cell cultures and drug treatment

MOLT-3 (CRL-1552^™^) cell line, which expresses the FP receptor [12], was purchased from the American Type Culture Collection (ATCC; Manassas, VA, USA). These cells were cultured in RPMI-1640 medium containing 2 mM L-glutamine, 2 g/l sodium bicarbonate, 4.5 g/l glucose, 10 mM HEPES, 1 mM sodium pyruvate (ATCC), and supplemented with 1% penicillin-streptomycin and 10% bovine calf serum (Invitrogen; Carlsbad, CA, USA). iHTM cells (gift from Zhongshan Ophthalmic Center of Sun Yat-sen University), generated as described [16], were cultured in Dulbecco’s modified Eagle’s medium (DMEM) containing 2.5 mM L-glutamine (ATCC) and supplemented with 1% penicillin-streptomycin and 10% bovine calf serum (Invitrogen).

Stock solutions of 100 mg/ml latanoprost and 100 mg/ml bimatoprost (Cayman Chemical; Ann Arbor, MI, USA) were prepared in dimethyl sulfoxide (DMSO). Three flasks of MOLT-3 cells were grown to about 30% confluence and treated daily with 0.1% DMSO as control, 10 μg/ml latanoprost or 10 μg/ml bimatoprost respectively [17], continuously for 5 days. The clinically used ester and amide drugs (latanoprost and bimatoprost respectively) were employed. After treatment, quantitative PCR (qPCR) analysis was performed to detect the effects of these PGAs on the expression level of mRNA (listed in Table 1). iHTM cells, cultured and treated as above, were maintained in 24-well plates. The cells were cultured to confluence or subconfluence for detecting the extracellular or intracellular distribution of fibronectin, respectively, by immunofluorescence.

**Table 1:** Primers of the specific genes for qPCR analysis.

Gene	Annealing temperature (°C)	Primers		Amplicon size (bp)	PCR efficiency (%)
		Forward	Reverse		
c-fos	59.6	GGCAAGGTGGAACAGTTAT	TCCGCTTGGAGTGTATCAG	125	95.2
TIMP-3	49.2	GCAACTCCGACATCGTGA	GCATCTTGGTGAAGCCTC	122	99.7
TIMP-4	57.0	ACGCCTTTTGACTCTTCC	GCAGCCACAGTTCAGATG	192	90.5
MMP-9	62.5	CGGAGCACGGAGACGGGTAT	CCGAGTTGGAACCACGACGC	146	100.9
fibronectin	62.2	CACTGTTTTGGTTCAGACTCG	AGATTTCCTCGTGGGCAGC	197	94.3
IкBα	56.2	AGCAGACTCCACTCCACTTG	GCCATTGTAGTTGGTAGCCT	218	95.0
NF-кBp65	56.2	CGCTGCATCCACAGTTTCCA	GTCCCCACGCTGCTCTTCTT	123	97.7
AQP1	62.5	CCTGGCTATTGACTACACTGG	CAGGATGAAGTCGTAGATGAGT	151	100.0
GAPDH	57.0	CCTCAAGATCATCAGCAAT	CCATCCACAGTCTTCTGGGT	141	97.0

### Preparation of RNA

Total RNA was isolated from the cultured MOLT-3 cells for each experimental condition with an extraction reagent (TRIzol Reagent; Invitrogen). RNA was prepared according to the protocol from the manufacturer. The RNA pellets were washed with 75% ethanol, centrifuged, air-dried and dissolved in RNase-free water. The residual DNA was removed by treatment with DNase I. Concentration and purity (i.e. OD_260_/OD_280_) of RNA samples were evaluated with a SmartSpec Plus spectrophotometer (Bio-Rad; Hercules, CA, USA) following the manufacturer’s instructions. RNA integrity was monitored by examining the 28S and 18S rRNA bands under UV light after separation on a 1% agarose gel and staining with GoldView I (Solarbio; Shanghai, China).

### qPCR assays

Total RNA (1 μg) was reverse-transcribed using a RevertAid^™^ first-strand cDNA synthesis kit (Fermentas; Vilnius, Lithuania) according to the manufacturer’s protocol. A 12 μl mixture containing 1 μg RNA, 1 μl oligo(dT)_18_ (0.5 μg/μl), and complementary RNase-free water was incubated at 65°C for 5 min, followed by adding 4 μl of the reaction buffer supplied with the kit, 1 μl RNase inhibitor (20 U/μl), 2 μl dNTP Mix (10mM), and 1 μl M-MuLv Reverse Transcriptase (200 U/μl). The total reaction system was incubated sequentially at 42°C for 60 min and 70°C for 5 min. qPCR was performed on iCycler-iQ5^®^ (Bio-Rad), with a total reaction mixture of 20 μl containing 1× SsoFast EVAGreen supermix (Bio-Rad), 2 μl of cDNA, and 330 nM of each of the forward and reverse primers. The cycling protocol consisted of one cycle of 30 s at 95°C, 40 cycles of 5 s at 95°C and 10 s at optimum annealing temperature (Table 1), followed by melt-curve data analysis. Fluorescence data were collected during the elongation step. Optimized primers were synthesized (HPLC purification grade) by Beijing Genomics Institute, China (Table 1). Assays for each RNA sample were performed in triplicate, and a standard curve from consecutive ten-fold dilutions of PCR products pool representative of a certain gene was included for PCR efficiency determination. Relative expression calculations, corrected with PCR efficiency and normalized with respect to the reference gene, glyceraldehyde-3-phosphate dehydrogenase (GAPDH, accession: NM 002046.3), were performed with IQ5 software (Bio-Rad). The results were expressed as the ratio of PCR-product/GAPDH mRNA. The data from three independent experiments with independent treatments and PCR reactions from each were reported as means*±*SD and used to calculate statistics.

### Immunofluorescence of cultured iHTM cells

To study the effects of PGAs on the extracellular and intracellular distribution pattern of fibronectin, immunofluorescence staining was performed on iHTM cells. For the former, iHTM cells were cultured to 100% confluence and fixed with 4% paraformaldehyde in phosphate-buffered saline (PBS, pH7.4) for 30 min, blocked with 10% goat serum in PBS overnight, and incubated with mouse anti-fibronectin monoclonal antibody (sc-18825, Santa Cruz Biotech; Paso Robles, CA, USA; 2 μg/ml) solution for 1 h. After three washes with PBS, the cells were incubated with goat anti-mouse IgG conjugated with fluorescein isothiocyanate (FITC, Zhongshan Goldenbridge Biotech; Beijing, China; 2 μg/ml) for 1 h at 37°C. After another three washes with PBS, the cells were viewed and photographed with a fluorescence microscope (Eclipse TE2000-U, Nikon Corp; Shanghai, China). A negative control was processed in the same manner except that the primary antibody was omitted. To study the intracellular distribution of fibronectin, the cells were cultured to about 30% confluence, then after 5 consecutive days of PGA treatment, permeabilized by 0.25% Triton X-100 in PBS for 5 min before fixation to facilitate antibody access to the cytoplasm.

Fluorescence intensity was measured using Image-pro Plus 6.0 (IPP6.0) System (Media Cybernetics; Rockville, MD, USA) to determine the means integrated option density to express the relative protein level. For each experimental condition, quantitative data originated from three independent experiments with independent treatments.

### Animal experiments

Six normotensive New Zealand white rabbits, weighing 2*±*0.5 kg, were housed under standard laboratory conditions of a 12-h light and 12-h dark cycle in individual cages with food and water *ad libitum*. Animal handling was in accordance with the ARVO Statement for the Use of Animals in Ophthalmic and Vision Research. Each group (three rabbits) received unilateral topical treatment with 30 μl of Xalatan (0.005% latanoprost; Pfizer, Karlsruhe, Germany) or Lumigan (0.03% bimatoprost; Pharm-Allergan, Ettlingen, Germany), administered in a masked fashion daily at 10:00 AM for 5 consecutive days. The contralateral eyes received normal saline (NS) only.

IOP was measured in conscious rabbits using a TonoVet™ rebound tonometer (Tiolat Oy, Helsinki, Finland) according to the manufacturer’s recommended procedures. The equipment was programmed to average the IOP values from six consecutive measurements. IOP was measured in a masked fashion immediately before each dosing, as well as 4 h and 8 h after dosing.

### Immunostaining of tissue sections

The rabbit eyes were enucleated 8 h after the last dosing, and fixed with 4% neutral buffered paraformaldehyde for 24 h at 4°C. After successive dehydration with 5% and 30% sucrose at 4°C overnight, the anterior segments of the eyes were excised and embedded in OCT Tissue Tek II embedding compound (Miles Scientific; Naperville, IL, USA). Cryostat sections (5 μm) were cut and air dried at room temperature for one day.

Immunostaining of the cryostat sections was performed the same way as for cultured cells. Briefly, after blocking, the cryostat sections were incubated successively in a moist chamber, with mouse monoclonal antibody to aquaporin1 (7D11; ab11025, Abcam; MA, USA; 2 μg/ml) as the primary antibody and goat anti-mouse IgG conjugated with FITC (2 μg/ml) as secondary antibody. A negative control was processed in the same manner except that the primary antibody was omitted. The immunostained cryostat sections were viewed and photographed with fluorescence microscope Eclipse TE2000-U. Fluorescence intensity was measured the same way as for cultured cells. For each experimental condition, quantitative data originated from three independent experiments with independent treatments.

### Statistical analysis

The experimental data were reported as *means±SD*. Each experiment was performed at least three times. Statistical comparison among the data was carried out using one-way analysis of variance (ANOVA) and SPSS 17.0 software (SPSS Inc.; Chicago, IL, USA). Two-tailed paired Student’s *t*-test was used to compare IOPs between drug- and vehicle-treated eyes. Differences were considered statistically significant when *p*<0.05.

## Results

### Effects of PGAs on the mRNA expression of MMPs

**Fig 1:**
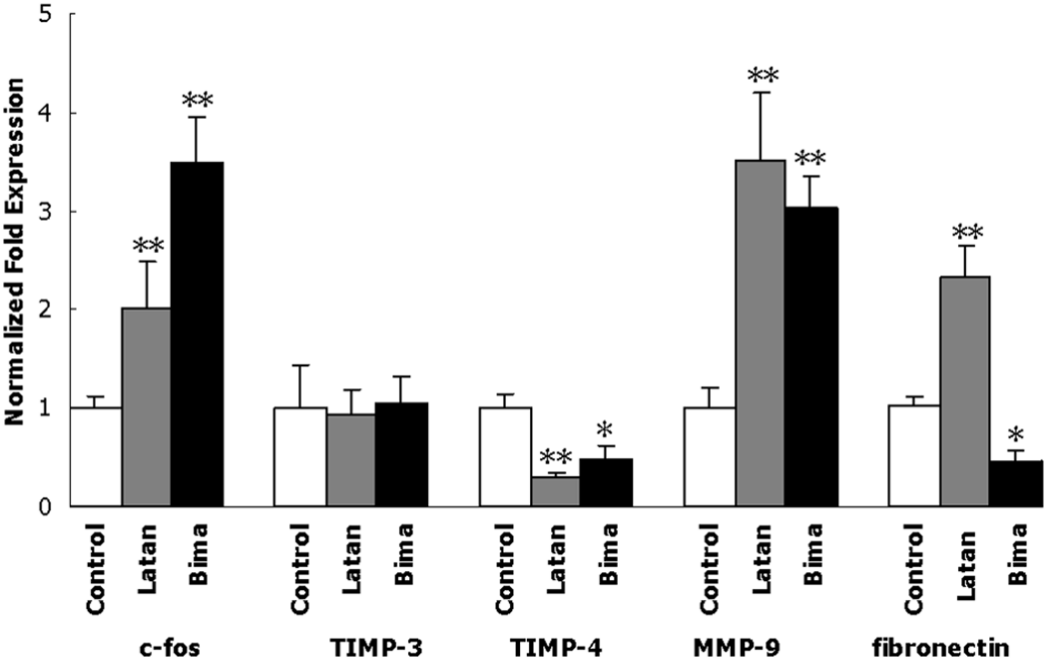
Effects of PGAs on the mRNA expression of fibronectin and ECM degradation related proteins in MOLT-3 cells after 5-day consecutive treatment. Control, MOLT-3 cells with 0.1% DMSO; Latan, MOLT-3 cells with 10 μg/ml lantanoprost; Bima, MOLT-3 cells with 10 μg/ml bimatoprost. Total RNA extracted from MOLT-3 cells was subjected to qPCR analysis and compared to that of the reference gene GAPDH as described in “Methods”. The expression value of control was considered as 1. Error bars represent *SD*, *n* = 3. *, *p*<0.05, **, *p*<0.01, versus control.

Degradation of extracellular matrix (ECM) in both conventional TM and uveoscleral aqueous humor outflow pathway, which derives from MMPs activation by PGA treatment, has been demonstrated [18–21]. To understand the effects of both latanoprost and bimatoprost, four genes, previously reported to be associated with the IOP-lowering effect of PGAs by inducing ECM degradation, were investigated in MOLT-3 cells using qPCR (Fig 1 and S1 Table). The mRNA of both c-fos and MMP-9 increased significantly with either latanoprost or bimatoprost. With either agent, there was little change in the mRNA expression of tissue inhibitor of metalloproteinase (TIMP)-3, but a significant decrease in TIMP-4.

### Effects of PGAs on fibronectin expression and degradation

**Fig 2:**
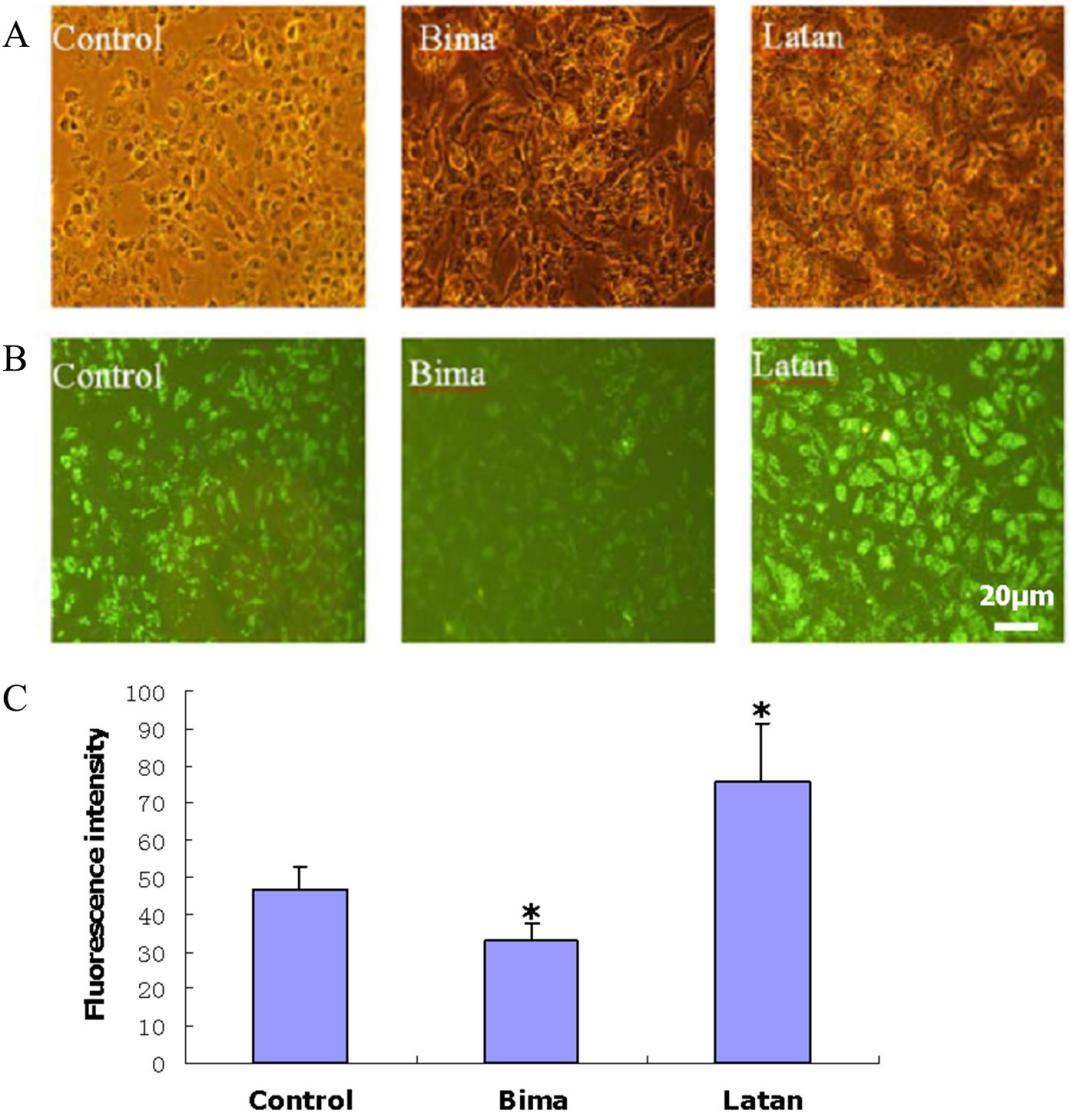
Changes in intracellular fibronectin levels in iHTM cells following PGA treatment. Before grown to confluence, cultures were treated with 0.1% DMSO (Control), 10 μg/ml bimatoprost (Bima) or 10 μg/ml latanoprost (Latan) continuously for 5 days. Cells were permeabilized and then labeled with antibody against fibronectin and IgG conjugated with FITC successively. A: Phase-contrast micrographs showing the morphology of iHTM cells treated with PGA or DMSO vehicle. B: Representative photomicrographs showing fibronectin immunostaining. Scale bar, 20 μm. C: Quantification of fluorescence intensity. Fluorescence intensity was measured using IPP 6.0 System to determine the means integrated option density to express the relative protein level. The results were expressed as *means±SD* (*n* = 3). *, *p*<0.05, compared with control.

**Fig 3:**
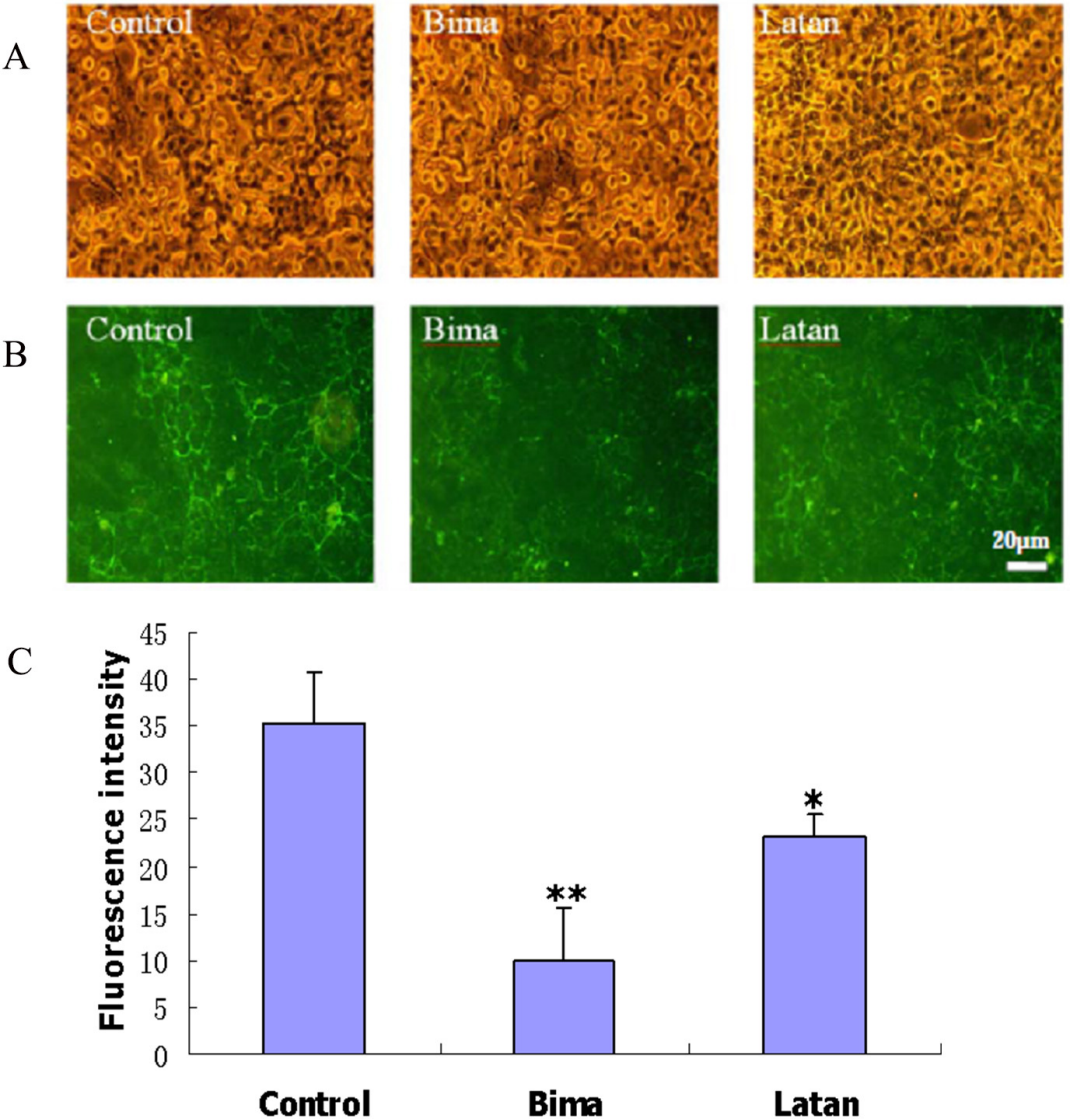
Decreased deposition of extracellular fibronectin in iHTM cells after PGA treatment. Confluent cell cultures were treated with 0.1% DMSO (Control), 10 μg/ml bimatoprost (Bima) or 10 μg/ml latanoprost (Latan) continuously for 5 days. Cells were then labeled with antibody against fibronectin and IgG conjugated with FITC successively. A: Phase-contrast micrographs showing the morphology of iHTM cells treated with PGA or vehicle DMSO. B: Representative photomicrographs showing fibronectin immunostaining. Scale bar, 20 μm. C: Quantification of fluorescence intensity. Fluorescence intensity was measured using IPP 6.0 System to determine the means integrated option density to express the relative protein level. The results were expressed as *means±SD* (*n* = 3). *, *p*<0.05, **, *p*<0.01, compared with control.

To better understand the IOP-lowering effect of PGAs by inducing ECM degradation, the fibronectin expression and breakdown, which would induce ECM turnover in the TM and change the resistance to aqueous humor outflow correspondingly [22–24], regulated by PGAs, was investigated. Treatment of MOLT-3 and iHTM cells for 5 consecutive days with either latanoprost or bimatoprost altered fibronectin expression at both the mRNA and protein levels. Fibronectin mRNA expression in MOLT-3 cells, determined by qPCR, was significantly up-regulated by latanoprost but was significantly down-regulated by bimatoprost (Fig 1 and S1 Table). Immunofluorescence analysis (Fig 2) of iHTM cell cultures indicated that intracellular fibronectin was also significantly increased by latanoprost (S1 and S2 Figs) and decreased by bimatoprost (S1 and S3 Figs). Immunostaining of extracellular fibronectin in control iHTM cell cultures revealed an extensive fibrous network (Fig 3 and S4 Fig). After treatment with PGAs, the fibronectin network in iHTM cells became discontinuous and less dense (Fig 3, S4, S5 and S6 Figs). The fibronectin network after bimatoprost treatment (S6 Fig) was significantly (*p<*0.05) less dense than that following latanoprost treatment (S5 Fig).

### Effects of PGAs on the mRNA expression of NF-κB signaling pathway

**Fig 4:**
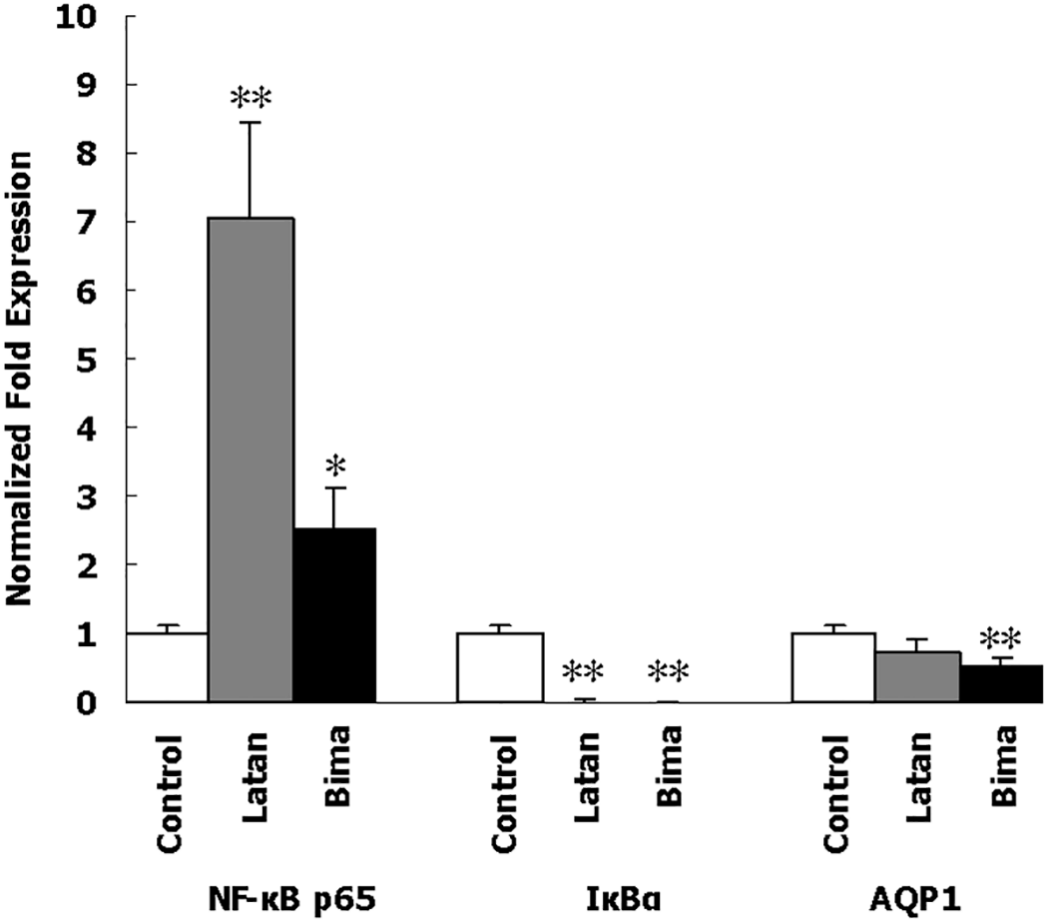
Effects of PGAs on NF-кB p65, IкBα and AQP1 mRNA expression in MOLT-3 cells after 5 days of continuous treatment. Control, MOLT-3 cells with 0.1% DMSO; Latan, MOLT-3 cells with 10 μg/ml lantanoprost; Bima, MOLT-3 cells with 10 μg/ml bimatoprost. Total RNA extracted from MOLT-3 cells was subjected to qPCR analysis and compared to that of the reference gene GAPDH as described in “Methods”. The expression value of control was considered as 1. Error bars represent *SD*, *n* = 3. *, *p*<0.05, **, *p*<0.01, versus control.

Besides the induction of c-fos, nuclear transcription factor NF-κB and its inhibitor IκB have also been found to be regulated by PGF_2_α [25]. To check the regulation on NF-κB signaling pathway by latanoprost and bimatoprost, the mRNA expression of NF-κB and IκB was also investigated. In MOLT-3 cells, there was a significant 7-fold increase in NF-кB p65 mRNA after treatment with latanoprost, and a significant 2.5-fold increase with bimatoprost (Fig 4 and S1 Table). The increase of NF-кB p65 mRNA with latanoprost was significantly higher than that with bimatoprost (*p<*0.05). The mRNA of IкBα, an inhibitor of NF-кB p65, decreased to nearly zero with either latanoprost or bimatoprost treatment (Fig 4 and S1 Table).

### Effects of PGAs on the mRNA and protein expression of AQP1

AQP1 involvement has also been reported in the IOP-lowering effects of PGAs [17]. In AQP1–knockout mice, IOP was decreased when compared to normal animals [26]. To further elucidate this proposition, AQP1 mRNA and protein expression after PGA treatment was investigated here in MOLT-3 cells and rabbit eyes separately.

Treatment of MOLT-3 cells for 5 consecutive days with either latanoprost or bimatoprost altered AQP1 expression at mRNA level. AQP1 mRNA expression, determined by qPCR, was insignificantly (*p =* 0.12758) down-regulated by latanoprost but was significantly down-regulated by bimatoprost (Fig 4 and S1 Table).

To test and verify the results obtained from MOLT-3 cells, we treated normotensive New Zealand white rabbits daily unilaterally with topical PGAs for 5 consecutive days, with NS administered contralaterally as vehicle, and then examined the AQP1 levels in the ciliary body tissues.

**Fig 5:**
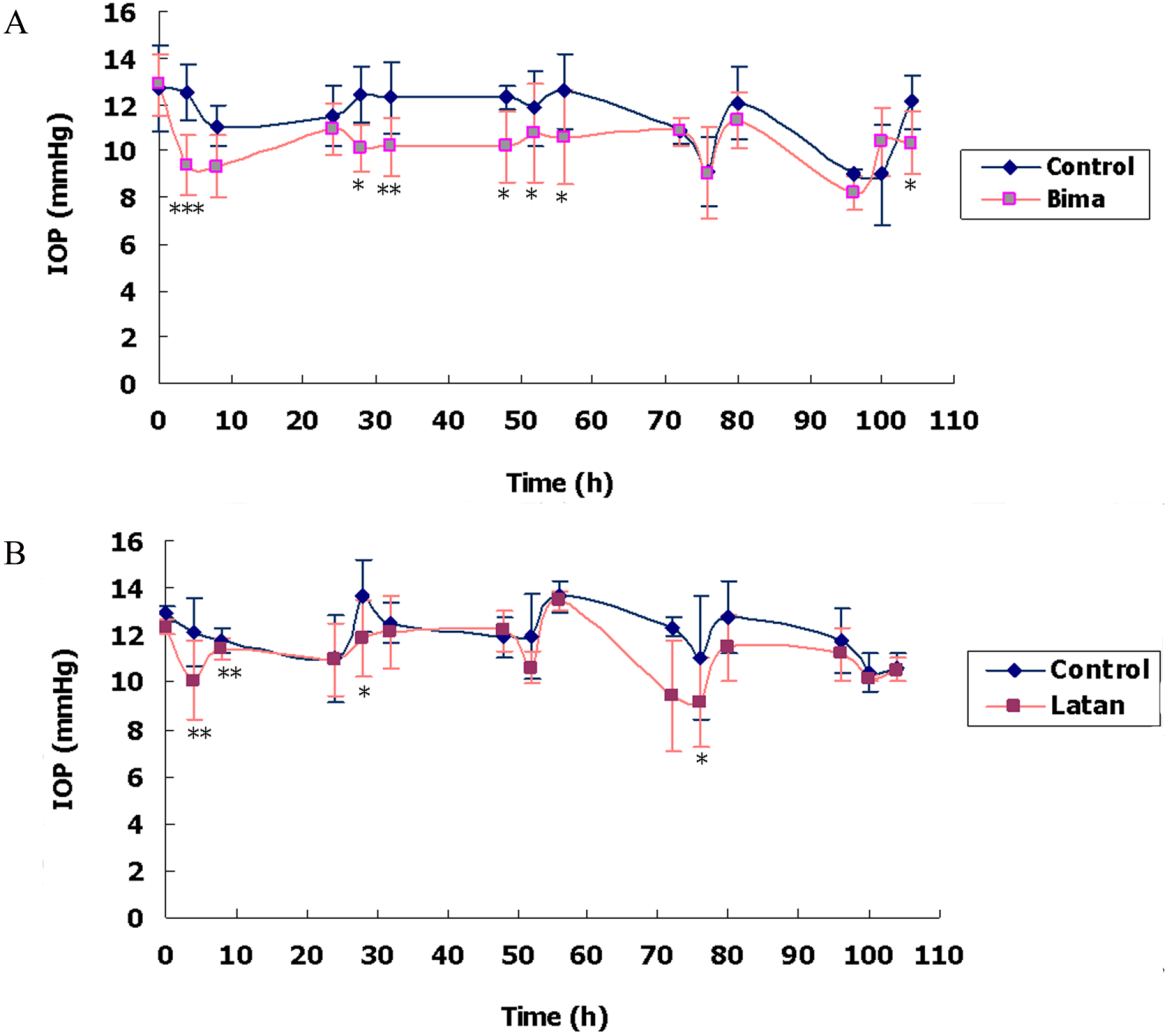
Effects of topical PGA medication on rabbit IOP. Thirty μl of Lumigan (Bima, A) or Xalatan (Latan, B) was administered unilaterally to rabbit eyes (qd) for 5 consecutive days, i.e. at 0, 24, 48, 72, and 96 h; NS (control) was administered contralaterally. IOP was measured in a masked fashion using a TonoVet^™^ rebound tonometer immediately before each dosing, as well as 4 h and 8 h after dosing. The results were expressed as *means±SD* (*n* = 3). *, *p*<0.05, **, *p*<0.01, ***, *p*<0.001, compared with control by two-tailed paired Student’s *t*-test.

Compared with NS, bimatoprost significantly lowered the IOP at 4 h and/or 8 h after daily drug administration (Fig 5A and S2 Table). On the 4^th^ day, the differences between IOP of bimatoprost eyes (i.e. 4 h after administration, 9.056*±*2.002 mmHg) and that of NS eyes (i.e. 4 h after administration, 9.111*±*1.456 mmHg) were insignificant, but both IOPs were significantly (*p<*0.01) lower than the baseline IOPs of the rabbit eyes (12.681*±*1.019 mmHg, *n =* 12). Systemic absorption of bimatoprost and circulation with blood may have reduced the IOP of contralateral NS eyes [27]. As with bimatoprost, latanoprost also lowered the IOP at 4 h and/or 8 h after daily drug delivery, although the reductions were relatively minor (Fig 5B and S2 Table).

**Fig 6:**
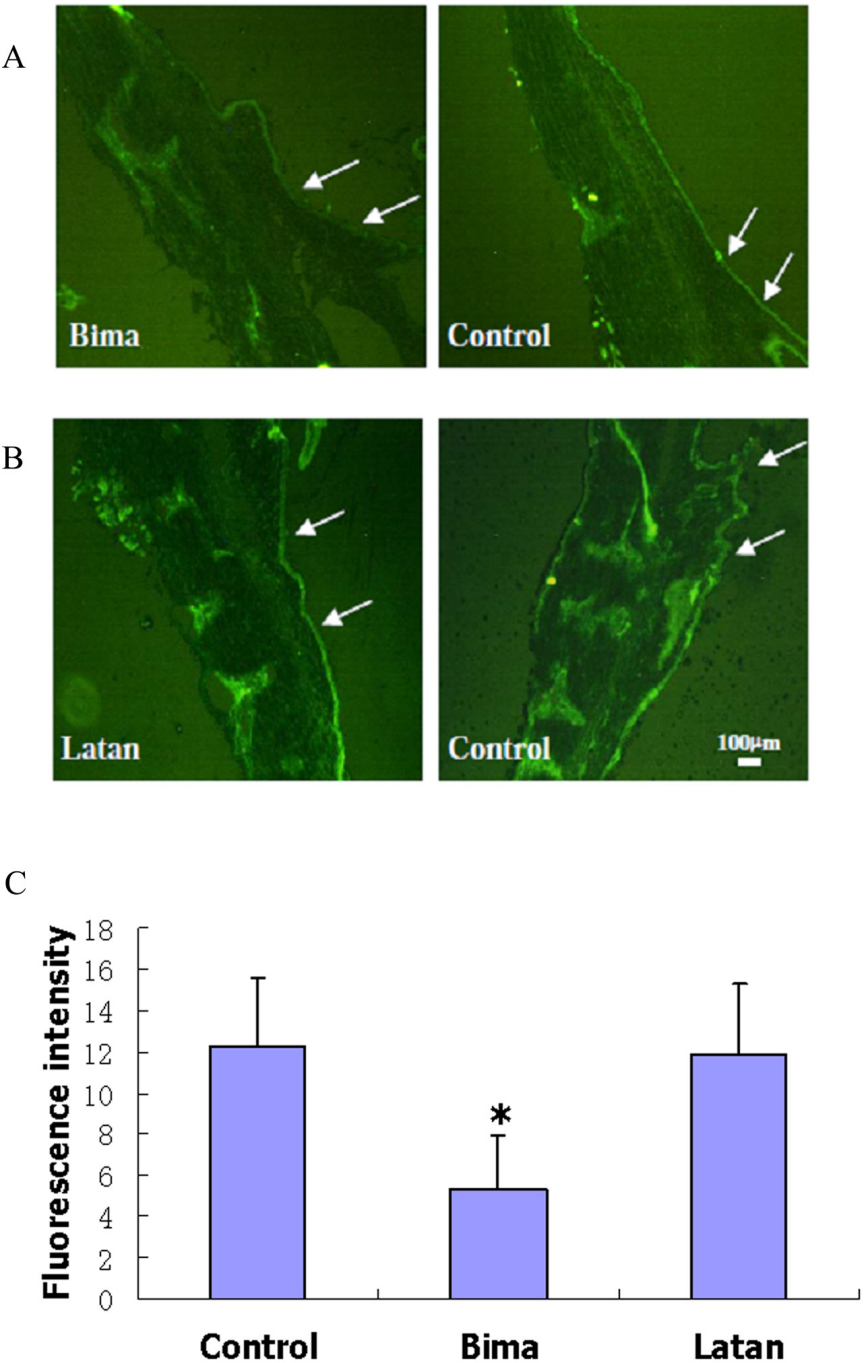
Effects of PGAs on AQP1 level in rabbit ciliary epithelium. Thirty μl of Lumigan (Bima, A) or Xalatan (Latan, B) was administered unilaterally to rabbit eyes (qd) continuously for 5 days; NS (control) was administered contralaterally. Rabbits were then sacrificed 8 h after the last dosing, and the eyes were enucleated, then ciliary body sections were prepared and immunostained with antibody against AQP1 and IgG conjugated with FITC. Arrows indicate the ciliary epithelium. Scale bar, 100 μm. (C) Quantification of fluorescence intensity. Fluorescence intensity was measured using IPP 6.0 System to determine the means integrated option density to express the relative protein level. The results were expressed as *means±SD* (*n* = 3). *, *p*<0.05, compared with control.

After five days of topical PGA treatment, immunostaining of the cryostat sections of rabbit eyes was performed with AQP1 antibody and IgG conjugated with FITC. The immunofluorescence of AQP1 was significantly less intense throughout the ciliary bodies of bimatoprost-treated eyes compared with those of controls (Fig 6A and 6C, S7 and S8 Figs). However, there was no visible difference in AQP1 immunofluorescence intensity between latanoprost-treated and control eyes (Fig 6B and 6C, S7 and S9 Figs).

## Discussion

Zhao et al [17] studied the effects of PGAs on human ciliary muscle (HCM) cells and human TM (HTM) cells, and concluded that both latanoprost and bimatoprost changed gene expression in HCM and HTM cells. We utilized the same dose (10 μg/ml) as Zhao to determine the changes following 5-day consecutive treatment with these drugs, although this dosage is high relative to the peak aqueous concentration after treatment with a therapeutic dose [peak aqueous concentration of latanoprost free acid of 78 nM (28 ng/ml)] [28]. With this concentration, in addition to the aqueous outflow related genes, Zhao et al explored several genes that have not been previously reported to be affected by these two PGAs, including AQP1 [17]. In the current study, we studied MOLT-3 cells with this dosage, hoping to find out if the differential effects of the different PGAs might involve differential changes of cellular proteins. We found that both bimatoprost and latanoprost affected the expression of various genes in MOLT-3 cells. The results that we expected with these cells were obtained, that is, the mRNA expression of both c-fos and MMP9 increased significantly in response to both agents in MOLT-3 cells, but AQP1 and fibronectin were differentially changed.

IOP is determined by the rate of aqueous humor production by the ciliary body and the drainage of aqueous humor through the TM and uveoscleral outflow pathways. In glaucomatous eyes, the increased resistance to aqueous humor outflow is likely due partly to increased ECM in the TM [15]. MMPs, neutral proteases that initiate degradation of ECM, are expressed by TM [29] and uveoscleral tissues [18–20, 30], and may play a major role in regulating outflow resistance [31, 32]. In this study, we found that latanoprost and bimatoprost had similar effects on the expression of MMP-9 and TIMP-4 genes, suggesting that they might both produce similar effects on ECM turnover to enhance outflow.

It is believed that PGA induction of MMPs is mediated by the activation of the proto-oncogene, c-fos [21, 30]. Lindsey et al reported that, in cultured HCM cells, PGA induced the expression of c-fos [21]. The response was maximal at 1 h after stimulation and was followed by a reduction to basal levels during the next several hours [21]. Our study showed that after 5 days of treatment with either latanoprost or bimatoprost in MOLT-3 cells, the mRNA level of c-fos still increased significantly. The difference between our results and those of Lindsey et al [21] might be due to the different doses (<0.2 μM was used in Lindsey et al) or durations of treatment (several hours were investigated in Lindsey et al). The immediate IOP effects that occur from a single dose of PGA may be mediated by cellular mechanisms different from those induced by repeated applications or continuous exposure [17, 32, 33].

NF-κB, one of the major transcription factors that play a critical role in the gene regulation of multiple cellular processes [34, 35], is also involved in MMP release [36, 37]. It was reported that down-regulation of IκBα, an inhibitor of NF-κB, resulted in decrease in TIMP-2 and increase in MMP-2 [37]. MMP-2 significantly increased outflow facility in a human eye outflow model [32]; furthermore, mechanical stress increased MMP-2 expression in cultured porcine TM cells [38]. Interestingly, latanoprost has been suggested to exert its effects on the remodeling of the ECM in the TM via MMP-2 and MMP-3 [39]. In this study, both latanoprost and bimatoprost were found to increase NF-кB and decrease IκBα. These results may suggest the potential involvement of NF-кB pathway in the IOP-lowering effect of PGAs. Yet, we must note that the changes of NF-кB and IκBα at the mRNA level should be confirmed at the protein level in the future.

Fibronectin expression and breakdown would induce ECM turnover in the TM and inner wall of Schlemm’s canal and change the resistance to aqueous humor outflow correspondingly [22–24]. In the present study, we found that fibronectin mRNA expression was up-regulated by latanoprost but down-regulated by bimatoprost. These divergent effects may suggest differing regulation of conventional TM aqueous flow resistance by bimatoprost and latanoprost. Besides the ECM (fibronectin) degradation by activated MMPs, the concurrently reduced fibronectin expression would help bimatoprost to reduce aqueous flow resistance. On the other hand, although the ECM (fibronectin) degradation was activated, the concurrently enhanced fibronectin expression might be a drag on latanoprost in reducing TM outflow resistance.

Yu et al investigated the effects of PGA drugs with benzalkonium chloride (BAC) as a preservative at 1:100 diluted commercial solutions, i.e. 0.00005% BAC in the final bimatoprost solution, and 0.0002% BAC in the final latanoprost solution, on fibronectin expression in HTM cells [40]. They found that fibronectin mRNA in the latanoprost-treated cells was 1.2*±*0.1 fold relative to control and in bimatoprost-treated cells was 0.7*±*0.2 fold. Since BAC at 1:100 diluted commercial solutions induced no effects on fibronectin expression [41], the changes were presumably induced by the PGAs. Although the changes were not statistically significant, possibly due to the short treatment duration (15 min), the directions of these changes are consistent with our current findings in MOLT-3 cells continuously treated for 5 days.

A similar result was also achieved by immunofluorescence in iHTM cells: intracellular fibronectin was significantly increased by latanoprost but decreased by bimatoprost. The extracellular fibronectin of bimatoprost-treated cultures was visualized as significantly less dense with a discontinuous fibrous network relative to that of latanoprost-treated cultures. The putatively greater effect of bimatoprost on the fibronectin network is likely due to its down-regulating the expression of fibronectin and up-regulating the expression of degradation enzymes such as MMPs. These results suggested that bimatoprost may be more effective than latanoprost in enhancing trabecular outflow. The IOP-lowering effects of bimatoprost might reflect a dual mechanism of action on aqueous humor outflow that involves both trabecular and uveoscleral pathways [42–44], even though its major effect is on the latter.

AQP water channels are small transmembrane proteins that function as passive conduits for osmotically or hydrostatically-driven water transport. AQP1 is expressed in the eye at sites of aqueous humor inflow and outflow, ciliary nonpigmented epithelium, inner (uveal and corneoscleral) and outer (juxtacanalicular) trabecular meshwork, plus Schlemm’s canal inner wall [45, 46]. The presence of AQP1 may provide these cells with high water permeability and allow water to be transported across these barriers efficiently [17]. In AQP1–knockout mice, IOP was decreased when compared to normal animals [26]. Thus, the action of PGAs in reducing expression of this gene may also help in decreasing IOP. Zhao et al reported that PGAs caused a decrease of AQP1 mRNA in HCM cells [17], which should cause less transmembrane transport of water across HCM cells. However, AQP1 deletion did not significantly affect aqueous outflow [26], suggesting that the transcellular pathway, mediated by AQP1, does not contribute significantly to the aqueous bulk outflow of human eyes [26, 47]. Additionally, uveoscleral outflow involves aqueous humor draining through the ECM between muscle bundles, not a pathway likely to dependent upon AQP1 unless it affects muscle contractility / relaxation.

Consistent with Zhao et al, our results showed that AQP1 expression was decreased in the ciliary epithelium in bimatoprost treated eyes. These studies suggested that AQP1 may play a role in IOP regulation and in the basis for IOP lowering effects of PGA, especially bimatoprost. However, the physiological significance of AQP1 regulation by PGAs is not well understood, and awaits further studies.

The IOP in any given eye is determined by the rate of aqueous production and the drainage of aqueous humor. In order to decrease IOP, most of the anti-glaucoma medications act either by decreasing the rate of aqueous humor production or by enhancing the aqueous outflow. The fact that the lower level of water permeability resulting from the reduced AQP1 expression can decrease IOP suggested a potential involvement of AQP1 in aqueous secretion across the ciliary epithelium [26]. However, a number of previous studies showed that PGAs were not able to decrease aqueous humor formation, and indeed might increase it slightly [15, 48]. We hypothesized that PGAs might also regulate other aqueous humor formation related proteins such as cystic fibrosis transmembrane conductance regulator [49, 50] or connexin43 [51, 52], which could counteract the effect of a reduction in AQP1.

## Conclusions

Latanoprost and bimatoprost both have been found in some studies to increase both uveoscleral outflow and conventional trabecular outflow facility [17, 31, 53, 54], but it is clear that for both drugs, the IOP reduction derives primarily from increasing unconventional (uveoscleral) outflow [15]. Based on our findings and other reports, both PGAs might produce similar effects on ECM turnover to enhance uveoscleral outflow. One potential reason for the small differences in IOP-lowering effect among the various PGAs could be their differing stimulating effects on trabecular outflow facility. Bimatoprost was thought to be more effective in lowering IOP than latanoprost because it enhanced conventional outflow facility by reducing ECM (fibronectin) expression. AQP1 may also play a role in the differential IOP regulation with various PGAs, possibly with aqueous humor formation affected.

## Supporting Information

S1 Fig Immunofluorescence analysis of intracellular fibronectin in control iHTM cell cultures. (RAR)

S2 Fig Immunofluorescence analysis of intracellular fibronectin in iHTM cell cultures after latanoprost treatment. (RAR)

S3 Fig Immunofluorescence analysis of intracellular fibronectin in iHTM cell cultures after bimatoprost treatment. (RAR)

S4 Fig Immunostaining of extracellular fibronectin in control iHTM cell cultures. (RAR)

S5 Fig Immunostaining of extracellular fibronectin in iHTM cell cultures after latanoprost treatment. (RAR)

S6 Fig Immunostaining of extracellular fibronectin in iHTM cell cultures after bimatoprost treatment. (RAR)

S7 Fig Immunostaining of AQP1 in control rabbit ciliary epithelium. (RAR)

S8 Fig Immunostaining of AQP1 in rabbit ciliary epithelium after bimatoprost treatment. (RAR)

S9 Fig Immunostaining of AQP1 in rabbit ciliary epithelium after latanoprost treatment. (RAR)

S1 Table qPCR analysis of mRNA expression in MOLT-3. (XLS)

S2 Table IOPs of rabbit eyes. (XLS)

## References

[1] Alexander CL, Miller SJ, Abel SR. Prostaglandin analog treatment of glaucoma and ocular hypertension. Ann Pharmacother 2002; 36:504–511. 11895065 10.1345/aph.1A178

[2] Aung T, Chew PT, Yip CC, Chan YH, See JL, Khng CG, et al A randomized double-masked crossover study comparing latanoprost 0.005% with unoprostone 0.12% in patients with primary open-angle glaucoma and ocular hypertension. Am J Ophthalmol 2001; 131:636–642. 11336940 10.1016/s0002-9394(00)00943-0

[3] Gandolfi SA, Cimino L. Effect of bimatoprost on patients with primary open-angle glaucoma or ocular hypertension who are nonresponders to latanoprost. Ophthalmology 2003; 110:609–614. 12623831 10.1016/S0161-6420(02)01891-2

[4] Scherer WJ. A retrospective review of non-responders to latanoprost. J Ocul Pharmacol Ther 2002; 18:287–291. 12099549 10.1089/108076802760116205

[5] Yu M, Chen XM, Ma J, Liu X. Travoprost and latanoprost, but not bimatoprost, induced nausea, vomiting and diarrhea. BMJ Case Reports. 2009; 10.1136/bcr.08.2008.0618 PMC3028105 21686721

[6] Simmons ST, Dirks MS, Noecker RJ. Bimatoprost versus latanoprost in lowering intraocular pressure in glaucoma and ocular hypertension: results from parallel-group comparison trials. Adv Ther 2004; 21:247–262. 15605619 10.1007/BF02850157

[7] Noecker RS, Dirks MS, Choplin NT, Bernstein P, Batoosingh AL, Whitcup SM. A six-month randomized clinical trial comparing the intraocular pressure-lowering efficacy of bimatoprost and latanoprost in patients with ocular hypertension or glaucoma. Am J Ophthalmol 2003; 135:55–63. 12504698 10.1016/s0002-9394(02)01827-5

[8] van der Valk R, Webers CA, Lumley T, Hendrikse F, Prins MH, Schouten JS. A network meta-analysis combined direct and indirect comparisons between glaucoma drugs to rank effectiveness in lowering intraocular pressure. J Clin Epidemiol 2009; 62:1279–1283. 10.1016/j.jclinepi.2008.04.012 19716679

[9] Cheng JW, Wei RL. Meta-analysis of 13 randomized controlled trials comparing bimatoprost with latanoprost in patients with elevated intraocular pressure. Clin Ther 2008; 30:622–632. 18498911 10.1016/j.clinthera.2008.04.006

[10] Ota T, Aihara M, Narumiya S, Araie M. The effects of prostaglandin analogues on IOP in prostanoid FP-receptor-deficient mice. Invest Ophthalmol Vis Sci 2005; 46:4159–4163. 16249494 10.1167/iovs.05-0494

[11] Chen J, Lu RT, Lai R, Dinh T, Paul D, Venadas S, et al Bimatoprost-induced calcium signaling in human T-cells does not involve prostanoid FP or TP receptors. Curr Eye Res 2009; 34:184–195. 10.1080/02713680802669781 19274525

[12] Li X, Liu G, Cai S, Guo L, Liu X. Human T lymphoblast cell line expresses FP receptor. Curr Eye Res 2011; 36:680–682. 10.3109/02713683.2011.566979 21599456

[13] Li X, Liu G, Wang Y, Yu W, Xiang H, Liu X. A case hypersensitive to bimatoprost and dexamethasone. J Ocul Pharmacol Ther 2011; 27:519–523. 10.1089/jop.2011.0036 21936632

[14] Cai S, Zhou X, Yan N, Liu X, Li X. Possible mechanism for the gastro-intestinal adverse effects upon topical application of prostaglandin F_2_α analogs. Med Hypotheses 2013; 80:32–35. 10.1016/j.mehy.2012.09.024 23098372

[15] Toris CB, Gabelt BT, Kaufman PL. Update on the mechanism of action of topical prostaglandins for intraocular pressure reduction. Surv Ophthalmol 2008; 53 Suppl1:S107–120. 10.1016/j.survophthal.2008.08.010 19038618 PMC2727743

[16] Polansky JR, Weinreb RN, Baxter JD, Alvarado J. Human trabecular cells. I. Establishment in tissue culture and growth characteristics. Invest Ophthalmol Vis Sci 1979;18:1043–1049. 383640

[17] Zhao X, Pearson KE, Stephan DA, Russell P. Effects of prostaglandin analogues on human ciliary muscle and trabecular meshwork cells. Invest Ophthalmol Vis Sci 2003; 44:1945–1952. 12714628 10.1167/iovs.02-0920

[18] Oh DJ, Martin JL, Williams AJ, Peck RE, Pokorny C, Russell P, et al Analysis of expression of matrix metalloproteinases and tissue inhibitors of metalloproteinases in human ciliary body after latanoprost. Invest Ophthalmol Vis Sci 2006; 47:953–963. 16505029 10.1167/iovs.05-0516

[19] Weinreb RN, Lindsey JD. Metalloproteinase gene transcription in human ciliary muscle cells with latanoprost. Invest Ophthalmol Vis Sci 2002; 43:716–722. 11867589

[20] Weinreb RN, Lindsey JD, Marchenko G, Marchenko N, Angert M, Strongin A. Prostaglandin FP agonists alter metalloproteinase gene expression in sclera. Invest Ophthalmol Vis Sci 2004; 45:4368–4377. 15557445 10.1167/iovs.04-0413

[21] Lindsey JD, To HD, Weinreb RN. Induction of c-fos by prostaglandin F2 alpha in human ciliary smooth muscle cells. Invest Ophthalmol Vis Sci 1994; 35:242–250. 8300352

[22] Floyd BB, Cleveland PH, Worthen DM. Fibronectin in human trabecular drainage channels. Invest Ophthalmol Vis Sci 1985; 26:797–804. 3891665

[23] Ueda J, Wentz-Hunter K, Yue BY. Distribution of myocilin and extracellular matrix components in the juxtacanalicular tissue of human eyes. Invest Ophthalmol Vis Sci 2002; 43:1068–1076. 11923248

[24] Babizhayev MA, Brodskaya MW. Fibronectin detection in drainage outflow system of human eyes in ageing and progression of open-angle glaucoma. Mech Ageing Dev 1989; 47:145–157. 2654504 10.1016/0047-6374(89)90017-1

[25] Taniguchi K, Matsuoka A, Kizuka F, Lee L, Tamura I, Maekawa R, et al Prostaglandin F2α (PGF2α) stimulates PTGS2 expression and PGF2α synthesis through NFKB activation via reactive oxygen species in the corpus luteum of pseudopregnant rats. Reproduction 2010; 140:885–892. 10.1530/REP-10-0240 20826536

[26] Zhang D, Vetrivel L, Verkman AS. Aquaporin deletion in mice reduces intraocular pressure and aqueous fluid production. J Gen Physiol 2002; 119:561–569. 12034763 10.1085/jgp.20028597 PMC2233864

[27] Tang-liu DD, Cherukury M, Acheampong A, Cheetham J, Vandenburgh A. Systemic pharmacokinetics of bimatoprost 0.03% solution following once daily ocular dosing in normal, healthy subjects. Arvo Annual Meeting Abstract Search & Program Planner Abstract 2002.

[28] Sjoquist B, Stjernschantz J. Ocular and systemic pharmacokinetics of latanoprost in humans. Surv Ophthalmol 2002; 47 Suppl 1:S6–12. 12204697 10.1016/s0039-6257(02)00302-8

[29] Alexander JP, Samples JR, Van Buskirk EM, Acott TS. Expression of matrix metalloproteinases and inhibitor by human trabecular meshwork. Invest Ophthalmol Vis Sci 1991; 32:172–180. 1846130

[30] Weinreb RN, Kashiwagi K, Kashiwagi F, Tsukahara S, Lindsey JD. Prostaglandins increase matrix metalloproteinase release from human ciliary smooth muscle cells. Invest Ophthalmol Vis Sci 1997; 38:2772–2780. 9418730

[31] Bahler CK, Howell KG, Hann CR, Fautsch MP, Johnson DH. Prostaglandins increase trabecular meshwork outflow facility in cultured human anterior segments. Am J Ophthalmol 2008; 145:114–119. 17988642 10.1016/j.ajo.2007.09.001 PMC2745953

[32] Bradley JM, Vranka J, Colvis CM, Conger DM, Alexander JP, Fisk AS, et al Effect of matrix metalloproteinases activity on outflow in perfused human organ culture. Invest Ophthalmol Vis Sci 1998; 39:2649–2658. 9856774

[33] Weinreb RN, Mitchell MD, Polansky JR. Prostaglandin production by human trabecular cells: in vitro inhibition by dexamethasone. Invest Ophthalmol Vis Sci 1983; 24:1541–1545. 6581147

[34] Ghosh S, May MJ, Kopp EB. NF-kappa B and Rel proteins: evolutionarily conserved mediators of immune responses. Annu Rev Immunol 1998; 16:225–260. 9597130 10.1146/annurev.immunol.16.1.225

[35] Baeuerle PA, Henkel T. Function and activation of NF-kappa B in the immune system. Annu Rev Immunol 1994; 12:141–179. 8011280 10.1146/annurev.iy.12.040194.001041

[36] Chase AJ, Bond M, Crook MF, Newby AC. Role of nuclear factor-kappa B activation in metalloproteinase-1, -3, and -9 secretion by human macrophages in vitro and rabbit foam cells produced in vivo. Arterioscler Thromb Vasc Biol 2002; 22:765–771. 12006388 10.1161/01.atv.0000015078.09208.92

[37] Lan YQ, Zhang C, Xiao JH, Zhuo YH, Guo H, Peng W, et al Suppression of IkappaBalpha increases the expression of matrix metalloproteinase-2 in human ciliary muscle cells. Mol Vis 2009; 15:1977–1987. 19816602 PMC2756517

[38] Bradley JM, Kelley MJ, Rose A, Acott TS. Signaling pathways used in trabecular matrix metalloproteinase response to mechanical stretch. Invest Ophthalmol Vis Sci 2003; 44:5174–5181. 14638714 10.1167/iovs.03-0213

[39] Ocklind A. Effect of latanoprost on the extracellular matrix of the ciliary muscle. A study on cultured cells and tissue sections. Exp Eye Res 1998; 67:179–191 9733584 10.1006/exer.1998.0508

[40] Yu AL, Fuchshofer R, Kampik A, Welge-Lussen U. Effects of oxidative stress in trabecular meshwork cells are reduced by prostaglandin analogues. Invest Ophthalmol Vis Sci 2008; 49:4872–4880. 10.1167/iovs.07-0984 18971427

[41] Guenoun JM, Baudouin C, Rat P, Pauly A, Warnet JM, Brignole-Baudouin F. In vitro comparison of cytoprotective and antioxidative effects of latanoprost, travoprost, and bimatoprost on conjunctiva-derived epithelial cells. Invest Ophthalmol Vis Sci 2005; 46:4594–4599. 16303954 10.1167/iovs.05-0776

[42] Brubaker RF, Schoff EO, Nau CB, Carpenter SP, Chen K, Vandenburgh AM. Effects of AGN 192024, a new ocular hypotensive agent, on aqueous dynamics. Am J Ophthalmol 2001; 131:19–24. 11162974 10.1016/s0002-9394(00)00843-6

[43] Christiansen GA, Nau CB, McLaren JW, Johnson DH. Mechanism of ocular hypotensive action of bimatoprost (Lumigan) in patients with ocular hypertension or glaucoma. Ophthalmology 2004; 111:1658–1662. 15350319 10.1016/j.ophtha.2004.02.006

[44] Wan Z, Woodward DF, Cornell CL, Fliri HG, Martos JL, Pettit SN, et al Bimatoprost, prostamide activity, and conventional drainage. Invest Ophthalmol Vis Sci 2007; 48:4107–4115. 17724194 10.1167/iovs.07-0080 PMC2680422

[45] Hamann S, Zeuthen T, La Cour M, Nagelhus EA, Ottersen OP, Agre P, et al Aquaporins in complex tissues: distribution of aquaporins 1–5 in human and rat eye. Am J Physiol 1998; 274:C1332–1345. 9612221 10.1152/ajpcell.1998.274.5.C1332

[46] Verkman AS. Role of aquaporin water channels in eye function. Exp Eye Res 2003; 76:137–143. 12565800 10.1016/s0014-4835(02)00303-2

[47] Stamer WD, Chan DW, Conley SM, Coons S, Ethier CR. Aquaporin-1 expression and conventional aqueous outflow in human eyes. Exp Eye Res 2008; 87:349–355. 10.1016/j.exer.2008.06.018 18657536 PMC2577040

[48] Woodward DF, Krauss AH, Nilsson SF. Bimatoprost effects on aqueous humor dynamics in monkeys. J Ophthalmol 2010; 2010:926192 10.1155/2010/926192 20508775 PMC2874926

[49] Yurko-Mauro KA, Reenstra WW. Prostaglandin F2alpha stimulates CFTR activity by PKA- and PKC-dependent phosphorylation. Am J Physiol 1998; 275:C653–660. 9730948 10.1152/ajpcell.1998.275.3.C653

[50] Levin MH, Verkman AS. Aquaporins and CFTR in ocular epithelial fluid transport. J Membr Biol 2006; 210:105–115. 16868675 10.1007/s00232-005-0849-1

[51] Calera MR, Wang Z, Sanchez-Olea R, Paul DL, Civan MM, Goodenough DA. Depression of intraocular pressure following inactivation of connexin43 in the nonpigmented epithelium of the ciliary body. Invest Ophthalmol Vis Sci 2009; 50:2185–2193. 10.1167/iovs.08-2962 19168903 PMC2865142

[52] Shirasuna K, Watanabe S, Nagai K, Sasahara K, Shimizu T, Ricken AM, et al Expression of mRNA for cell adhesion molecules in the bovine corpus luteum during the estrous cycle and PGF2alpha-induced luteolysis. J Reprod Dev 2007; 53:1319–1328. 17827879 10.1262/jrd.19082

[53] Toris CB, Camras CB, Yablonski ME, Brubaker RF. Effects of exogenous prostaglandins on aqueous humor dynamics and blood-aqueous barrier function. Surv Ophthalmol 1997; 41:S69–75. 9154279 10.1016/s0039-6257(97)80010-0

[54] Weinreb RN, Toris CB, Gabelt BT, Lindsey JD, Kaufman PL. Effects of prostaglandins on the aqueous humor outflow pathways. Surv Ophthalmol 2002; 47:S53–64. 12204701 10.1016/s0039-6257(02)00306-5

